# Common mental disorders in São Paulo city, 2003-2015: population-based serial cross-sectional panel studies

**DOI:** 10.1590/S2237-96222025v34.20240048.en

**Published:** 2025-05-09

**Authors:** Thiago Pestana Pinto, Camila Nascimento Monteiro, Moises Goldbaum, Chester Luiz Galvão César, Paulo Rossi Menezes

**Affiliations:** 1 Universidade de São Paulo, Faculdade de Saúde Pública, São Paulo, SP, Brazil; 2 Hospital Sírio-Libanês, São Paulo, SP, Brazil; 3 Universidade de São Paulo, Faculdade de Medicina, São Paulo, SP, Brazil

**Keywords:** Mental Disorders, Mental Health, Health Surveys, Serial Cross-Sectional Studies, Epidemiology, Trastornos Mentales, Salud Mental, Encuestas Epidemiológicas, Estudios Transversales Seriados, Epidemiología

## Abstract

**Objective:**

To describe the temporal evolution of common mental disorders among individuals aged 20 or over living in the urban area of ​​São Paulo city.

**Methods:**

Population-based serial cross-sectional panel studies with data from the 2003, 2008 and 2015 editions of the São Paulo City Health Survey. Presence of common mental disorders was measured using the Self-Reporting Questionnaire-20. We used Poisson regression to estimate crude and adjusted prevalence ratios (PR) and 95% confidence intervals (95%CI) to verify differences in the prevalence of common mental disorders between years.

**Results:**

The total number of participants in this investigation was 1,565 in 2003, 2,019 in 2008 and 3,169 in 2015. In the adjusted analysis, there was a reduction in the prevalence of common mental disorders between 2003 and 2015 in females aged between 20 and 59 years old (PR 0.68; 95%CI 0.56; 0.84). In the same period, there was no significant variation among male individuals aged 20 to 59 (PR 1.25; 95%CI 0.86; 1.82), nor among females aged 60 or over (PR 0.95; 95%CI 0.69; 1.32) and males aged 60 or over (PR 0.68; 95%CI 0.44; 1.07).

## Resumen

**Objetivo:**

Describir la evolución temporal de los trastornos mentales comunes entre personas de 20 años o más residentes en el área urbana de la ciudad de São Paulo.

**Métodos:**

Panel de estudios transversales seriados de base poblacional con datos de las ediciones de 2003, 2008 y 2015 de la Encuesta de Salud del Municipio de São Paulo. La presencia de trastornos mentales comunes se midió mediante el Cuestionario de Autoinforme de 20 Ítems (SRQ-20). Utilizando la regresión de Poisson, se estimaron las razones de prevalencia (RP) no ajustadas y ajustadas y los intervalos de confianza del 95% (IC95%) para verificar las diferencias en la prevalencia de los trastornos mentales comunes entre años.

**Resultados:**

El número total de participantes en esta investigación fue de 1.565 en 2003, 2.019 en 2008 y 3.169 en 2015. En el análisis ajustado, hubo una reducción en la prevalencia de trastornos mentales comunes entre 2003 y 2015 en mujeres de entre 20 y 59 años. (RP 0,68; IC95% 0,56; 0,84). En el mismo período, no hubo variación significativa entre los hombres de 20 a 59 años (RP 1,25; IC95% 0,86; 1,82), así como entre las mujeres de 60 años o más (RP 0,95; IC95% 0,69; 1,32) y los hombres de 60 años o más (RP 0,68; IC95% 0,44; 1,07).

**Conclusión:**

Entre 2003 y 2015, la prevalencia de trastornos mentales comunes en la ciudad de São Paulo disminuyó en mujeres con edades entre 20 y 59 años, mientras se mantuvo estable en los demás subgrupos analizados. Estos hallazgos muestran la importancia de las acciones de salud mental y el seguimiento de la prevalencia de esta morbilidad psiquiátrica en la población, considerando diferencias por sexo y grupo de edad.

## Introduction

Mental health problems are one of the main causes of disability worldwide (1). Among these health problems, common mental disorders, characterized by a set of depressive symptoms, anxiety and somatic complaints, constitute a frequent psychiatric morbidity that, although it does not necessarily meet criteria defined by diagnostic manuals for a specific mental disorder, can also strongly impact functional capacity (2).

In Brazil, population studies in different urban centers have reported high prevalence of these disorders, varying between 15% and 28% (3-5). Despite this evidence, there is a gap in data on the temporal evolution of the prevalence of this morbidity.

Considering the pre-COVID-19 pandemic period, from 2001 to 2019, divergent results have been reported in trend analyses of mental health problems, such as depressive disorders, anxiety disorders, psychological distress and other disorders (6-12). For example, while in Brazil (12) an increase in prevalence of depressive symptoms was identified between 2013 and 2019, in Peru (10) prevalence remained stable between 2014 and 2018. In Spain, between 2006 and 2016, a reduction was seen in common mental disorders among women and an increase among younger men. This information is less accurate for low- and middle-income countries, due to the limited number of serial population-based epidemiological studies addressing the topic in these regions, as shown in previous systematic reviews (13,14).

Investigating the distribution of mental disorders over time can provide relevant information for planning and monitoring mental health actions. This study aimed to describe the temporal evolution (2003-2015) of the prevalence of common mental disorders in adults living in the urban area of ​​São Paulo city.

## Methods

### Design

This is a population-based serial cross-sectional panel study, conducted using data collected in the 2003, 2008 and 2015 editions of the São Paulo City Health Survey (*Inquérito de Saúde do Município de São Paulo* - ISA-Capital).

### Background

Conducted periodically, the ISA-Capital Survey investigates aspects related to living conditions, health and use of health services among the population residing in the urban area of ​​São Paulo city (15).

### Participants

This study included individuals aged 20 or over who answered the mental health block of questions. The individuals were selected in the three editions of the ISA-Capital Survey by means of cluster sampling in two stages: census tracts and households. A total of 60 census tracts were randomly selected in 2003, 70 in 2008 and 150 in 2015. Response rates were similar in all three years: 75% in 2003, 76% in 2008 and 74% in 2015. In order to correct for response effects, percentage losses of 20% in 2003 and 2008, and 26% in 2015 were taken into consideration. As the target population of this investigation was adults, we only used data referring to the demographic domains for those who were 20-59 years old and 60 or over from the three years of the survey.

### Variables

The outcome was the presence of common mental disorders, assessed using the Self-Reporting Questionnaire-20 (16), cited in the mental health block of the questionnaires administered in the three years of the ISA-Capital Survey. Considering a more recent performance evaluation (17) of this instrument in adults, which presented sensitivity of 86% and specificity of 89%, we adopted a cut-off point of 8 affirmative responses for both sexes, that is, participants who answered “yes” to 8 or more of the 20 items were considered as having common mental disorders.

The following sociodemographic variables were also used: sex (male, female); age group (in years: 20-59 and ≥60); race/skin color (White, non-White); marital status (married or stable union, single, separated or divorced, widowed); schooling (in years:≤3, 4-11, ≥12); monthly family income per capita (in minimum wages <1, 1-4 and >4); and paid or voluntary work (yes, no).

### Data source and measurement

Data collection for each survey was carried out by means of questionnaires structured into thematic blocks, administered during interviews in the participants’ homes.

### Bias control

The interviewers were trained beforehand and were monitored throughout the fieldwork period. In order to check the quality of the data in each year of the survey, additional interviews were carried out, by telephone or at home, in a random sample comprised of 5% of the participants.

### Study size

The sample size calculation in the three editions of the ISA-Capital Survey took into account a scenario equivalent to the maximum variability in the frequency of the events studied (proportion of 0.50), with a design effect of 1.5 and a 95% confidence level. The sampling error was 6% in 2003 and 2008, and 10% in 2015. In 2003 and 2008, eight demographic domains were considered in the total sample, namely: <1 year old; 1-11 years old; 12-19 years old, male and female; 20-59 years old, male and female; and ≥60 years old, male and female. In the 2015 ISA-Capital Survey, the domains were: 12-19 years old, male and female; 20-59 years old, male and female; and ≥60 years old, male and female. The final samples were 3,357 in 2003, 3,271 in 2008 and 4,043 in 2015. When considering only individuals aged 20 or over, the sample was 1,667 in 2003, 2,086 in 2008 and 3,169 in 2015. Additional information on methodological aspects of the three editions of the ISA-Capital Survey can be consulted in previous publications (15,18).

### Statistical methods

Descriptive analyses were carried using the sociodemographic variables. The general and sex-stratified prevalence of common mental disorders were estimated both for the total sample and by age (20-59 and ≥60 years old), for each year of the survey.

Analysis of temporal evolution was performed by comparing the prevalence of common mental disorders in both the general population and also in the sex and age group subgroups, between 2003 and 2008, as well as between 2003 and 2015. To this end, the names and categories of the variables were standardized in the original survey databases. Following this, two unified databases were created, one for 2003 and 2008 and the other for 2003 and 2015, taking into consideration the sampling process of the different years in the adjustment of the sampling weights in these unified databases.

In order to analyze the magnitude of differences in prevalence of common mental disorders between years, crude and adjusted prevalence ratios (PR) and 95% confidence intervals (95%CI) were estimated using Poisson regression models. The PRs were adjusted for age, sex, race/skin color, marital status, schooling, income and work to verify whether the differences in the prevalence of common mental disorders, between the editions of the survey, were independent of the other variables of the sociodemographic structure. In models stratified by sex, adjustments were made for the age, race/skin color, marital status, schooling, income and work variables.

The behavior of the difference in the prevalence of common mental disorders between sexes in the total population and by age group (20-59 and ≥60 years) was analyzed. The magnitude of this difference was presented through crude and adjusted PRs for age, race/skin color, marital status, schooling, income and work, for each year of the survey.

We calculated interaction between the sex and survey year variables, in models adjusted by survey year, age, sex, race/skin color, marital status, schooling, income and work, and the p-values ​​of these interaction analyses were calculated.

Statistical significance was assessed using the Wald test, adopting a 5% significance level. Observations with missing data were excluded, and the analyses were performed with Stata 13 using the svy module for data obtained through complex sampling.

## Results

In all, this study included 1,565 individuals in 2003, 2,019 in 2008 and 3,169 in 2015. The percentage distribution of sex (m/f) between the years remained stable, with the majority being female (54.9% in 2003 versus 53.7% in 2015). There was an increase in the proportion of people aged 60 or over (16.0% in 2003 vs. 18.5% in 2015). There was a decrease in the proportion of people who self-reported their race/skin color as White (67.4% in 2003 vs. 52.7% in 2015) and an increase in the lower income bracket (38.2% in 2003 vs. 54.5% in 2015) (Table 1).

**Table 1:** Frequencies and 95% confidence intervals (95%CI) of adult sociodemographic characteristics by survey year. São Paulo, 2003 (n=1,667), 2008 (n=2,086) and 2015 (n=3,184)

Variables	n	2003, % (95%CI)	n	2008, % (95%CI)	n	2015, % (95%CI)
Sex						
Male	803	45.1 (42.1; 48.1)	848	46.3 (44.3; 48.3)	1,340	46.3 (44.6; 48.0)
Female	864	54.9 (51.9; 57.9)	1,238	53.7 (51.7; 55.7)	1,844	53.7 (52.0; 55.4)
**Age group**						
20-59	795	84.0 (81.6; 86.2)	1,162	83.7 (81.2; 85.9)	2,165	81.5 (79.3; 83.5)
≥60	872	16.0 (13.8; 18.4)	924	16.3 (14.1; 18.8)	1,019	18.5 (16.5; 20.7)
**Race/skin color**						
White	1,077	67.4 (61.7; 72.6)	1,311	62.2 (57.2; 66.9)	1,632	52.7 (48.8; 56.5)
Non-white	542	32.6 (27.4; 38.3)	770	37.8 (33.1; 42.8)	1,531	47.3 (43.5; 51.2)
**Marital status**						
Married or stable union	969	58.1 (54.3; 61.8)	1,181	59.4 (56.3; 62.5)	1,820	59.0 (56.5; 61.6)
Single	272	27.0 (23.8; 30.5)	390	25.4 (22.6; 28.4)	712	25.9 (23.9; 28.0)
Separated or divorced	124	7.3 (5.83; 9.21)	187	8.3 (6.80; 10.1)	308	8.7 (7.60; 9.91)
Widowed	274	7.6 (6.24; 9.18)	328	6.9 (5.80; 8.23)	333	6.4 (5.57; 7.32)
**Schooling** (years)						
≥12	231	23.9 (18.1; 30.9)	305	24.2 (19.3; 29.9)	686	28.6 (24.8; 32.7)
4-11	959	61.9 (57.0; 66.6)	1,365	65.6 (61.4; 69.6)	1,935	59.7 (56.2; 63.1)
≤3	450	14.2 (11.4; 17.5)	413	10.2 (8.24; 12.6)	545	11.7 (10.2; 13.4)
**Family income** (**minimum wages**)						
>4	233	19.5 (13.0; 28.3)	219	14.8 (10.6; 20.2)	254	8.9 (7.04; 11.3)
1-4	745	42.3 (37.1; 47.7)	1,023	47.5 (43.2; 51.8)	1,141	36.6 (32.8; 40.5)
<1	689	38.2 (32.0; 44.7)	844	37.7 (32.7; 43.1)	1,789	54.5 (50.2; 58.7)
**Paid/voluntary work**						
Yes	654	57.5 (54.1; 60.9)	975	65.3 (62.3; 68.1)	1,787	64.2 (61.9; 66.5)
No	995	42.5 (39.1; 45.9)	1,110	34.7 (31.9; 37.7)	1,385	35.8 (33.5; 38.1)

In 2003, overall prevalence of common mental disorders was 22.0% (95%CI 18.8; 25.7); there was a significant reduction both in 2008 (PR 0.76; 95%CI 0.61; 0.94) and in 2015 (PR 0.76; 95%CI 0.62; 0.93). After adjustment for the set of sociodemographic structure variables, these differences remained statistically significant, both in the period 2003-2008 (PR 0.81; 95%CI 0.67; 0.97) and in the period 2003-2015 (PR 0.80; 95%CI 0.67; 0.95).

Among those between 20 and 59 years old, significant differences were found for both sexes in the prevalence of common mental disorders, between 2003 and 2008 (PR 0.80; 95%CI 0.65; 0.97) and between 2003 and 2015 (PR 0.79; 95%CI 0.65; 0.96). Among the elderly, no significant changes were observed in the prevalence of disorders between 2003 and 2008 (PR 0.86; 95%CI 0.70; 1.05) and between 2003 and 2015 (PR 0.89; 95%CI 0.67; 1.18) (Table 2).

**Table 2:** Prevalence, prevalence ratios (PR) and 95% confidence intervals (95%CI) of common mental disorders by sex, age group and survey year. São Paulo, 2003 (n=1,565), 2008 (n=2,019) and 2015 (n=3,169)

Age group and sex	2003	2008	2015	2003-2008				2003-2015			
	% (95%CI)	% (95%CI)	% (95%CI)	Crude PR (95%CI)	p-value	Adjusted PR (95%CI)	p-value	Crude PR (95%CI)	p-value	Adjusted PR (95%CI)	p-value
≥**20 years**											
Total	22.0 (18.8; 25.7)	16.8 (14.9; 18.8)	16.8 (15.3; 18.5)	0.76 (0.61; 0.94)	0.012	0.81 (0.67; 0.97)	0.021	0.76 (0.62; 0.93)	0.009	0.8 (0.67; 0.95)	0.011
Male	9.8 (8.7; 9.2)	8.0 (6.0; 10.6)	10.4 (8.8; 12.4)	0.82 (0.56; 1.21)	0.322	0.86 (0.60; 1.25)	0.439	1.07 (0.78; 1.47)	0.672	1.14 (0.83; 1.56)	0.42
Female	32 (27.2; 37.1)	24.3 (21.5; 27.2)	22.4 (20.0; 24.9)	0.75 (0.60; 0.93)	0.011	0.80 (0.66; 0.96)	0.017	0.70 (0.57; 0.86)	0.001	0.72 (0.60; 0.87)	0.001
**20-59 years**											
Total	22.3 (18.6; 26.6)	16.6 (14.6; 18.9)	16.8 (15.2; 18.7)	0.74 (0.59; 0.94)	0.016	0.80 (0.65; 0.97)	0.026	0.75 (0.60; 0.94)	0.014	0.79 (0.65; 0.96)	0.018
Male	8.8 (6.3; 12.1)	7.8 (5.6; 10.8)	10.6 (8.8; 12.8)	0.89 (0.56; 1.40)	0.606	0.92 (0.59; 1.43)	0.703	1.21 (0.83; 1.75)	0.324	1.25 (0.86; 1.82)	0.235
Female	33.6 (28.1; 39.7)	24.5 (21.5; 27.8)	22.5 (19.9; 25.4)	0.73 (0.58; 0.92)	0.008	0.77 (0.63; 0.94)	0.012	0.67 (0.53; 0.84)	0.001	0.68 (0.56; 0.84)	<0.001
≥**60 years**											
Total	20.5 (17.4; 23.9)	17.4 (15.0; 20.1)	16.8 (13.4; 20.8)	0.85 (0.68; 1.06)	0.150	0.86 (0.70; 1.05)	0.132	0.82 (0.62; 1.08)	0.152	0.89 (0.67; 1.18)	0.405
Male	15.6 (11.5; 20.9)	9.3 (6.2; 13.6)	9.5 (6.8; 13.0)	0.59 (0.36; 0.98)	0.041	0.62 (0.39; 1.01)	0.054	0.60 (0.39; 0.95)	0.028	0.68 (0.44; 1.07)	0.095
Female	23.7 (19.2; 28.9)	23.1 (19.4; 27.2)	21.7 (17.0; 27.4)	0.97 (0.74; 1.27)	0.846	0.91 (0.74; 1.20)	0.621	0.82 (0.67; 1.26)	0.594	0.95 (0.69; 1.32)	0.778

**Figure 1:**
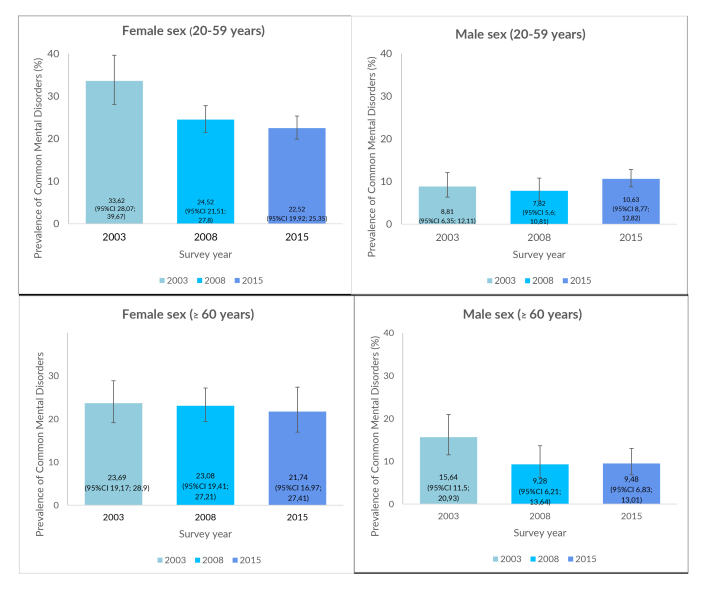
Prevalence and 95% confidence intervals (95%CI) of common mental disorders by sex, age group and survey year. São Paulo, 2003 (n=1,565), 2008 (n=2,019) and 2015 (n=3,169)

Among male participants in the 20-59 age group, there was no significant variation in the prevalence of common mental disorders between 2003 and 2008 (PR 0.92; 95%CI 0.59; 1.43) and between 2003 and 2015 (PR 1.25; 95%CI 0.86; 1.82). Likewise, no significant changes were found among elderly men in the periods 2003-2008 (PR 0.62; 95%CI 0.39; 1.01) and 2003-2015 (PR 0.68; 95% CI 0.44; 1.07). Among women aged 20 to 59 years, there was a significant reduction in the prevalence of common mental disorders both between 2003 and 2008 (PR 0.77; 95%CI 0.63; 0.94) and also between 2003 and 2015 (PR 0.68; 95%CI 0.56; 0.84). On the other hand, among elderly women, no changes were identified in the periods 2003-2008 (PR 0.91; 95%CI 0.74; 1.20) and 2003-2015 (PR 0.95; 95 %CI 0.69; 1.07) (Table 2) e (Figure 1).

Especially in the 20-59 age group, there was a reduction in the magnitude of the difference in the prevalence of common mental disorders between the sexes, going from PR 3.19 (95%CI 2.31; 4.42) in 2003 to PR 2.01 (95%CI 1.58; 2.55) in 2015. Considering the 2003 and 2008 editions of the ISA-Capital Survey, there was no significant interaction between sex and survey year either in the total population (p-value 0.901), or in the 20-59 age group (p-value 0.606) and the 60 or over age group (p-value 0.155). In the comparison between 2003 and 2015, the temporal evolution of common mental disorders was significantly different between sexes in the general population (p-value 0.015) and in the 20-59 age group (p-value 0.005) (Table 3).

**Table 3:** Prevalence ratios (PR) and 95% confidence intervals (95%CI) of common mental disorders between the sexes, by age group and survey year. São Paulo, 2003 (n=1,565), 2008 (n=2,019) and 2015 (n=3,169)

Age group and sex	2003		2008		2015		Interaction sex##year	
	crude PR (95%CI)	adjusted PR (IC 95%)	crude PR (95%CI)	adjusted PR (IC 95%)	crude PR (95%CI)	adjusted PR (IC 95%)	2003-2008	2003-2015
≥**20 years**								
Male	1.00	1.00	1.00	1.00	1.00	1.00	0.901	0.015
Female	3.28 (2.53; 4.23)	2.78 (2.12; 3.64)	3.03 (2.26; 4.05)	2.88 (2.08; 4.01)	2.14 (1.75; 2.62)	2.02 (1.63; 2.51)		
**20-59 years**								
Male	1.00	1.00	1.00	1.00	1.00	1.00	0.606	0.005
Female	3.81 (2.81; 5.18)	3.19 (2.31; 4.42)	3.13 (2.22; 4.43)	3.02 (2.03; 4.50)	2.12 (1.69; 2.65)	2.01 (1.58; 2.55)		
≥**60 years**								
Male	1.00	1.00	1.00	1.00	1.00	1.00	0.155	0.307
Female	1.51 (1.03; 2.23)	1.44 (0.89; 2.30)	2.49 (1.59; 3.89)	2.03 (1.20; 3.42)	2.29 (1.62; 3.25)	1.85 (1.23; 2.78)		

## Discussion

Between 2003 and 2015, a significant reduction in the prevalence of common mental disorders was found in women up to 59 years of age. On the other hand, prevalence did not change among men, nor among the elderly. Interaction between the sex and survey year variables in the 20-59 age group indicates that, between 2003 and 2015, the temporal evolution of common mental disorder prevalence was different between the sexes, with a significant decrease in prevalence among women in comparison to men.

Unlike this investigation, an increase in the prevalence of depressive symptoms was found between 2013 and 2019 in the National Health Survey (*Pesquisa Nacional de Saúde*) (12). The economic recession in Brazil, in 2015 and 2016, is an important factor to be considered in the comparison with our investigation. As emphasized by the authors of the National Health Survey, much of the period analyzed by them was permeated by economic slowdown and recession in Brazil, which would explain this upward trend (12).

In turn, also in South America, a health survey carried out in Chile reported stable prevalence of depressive disorders between 2003 and 2010 in individuals aged 18 or over. A similar result was identified by a survey in Peru, which reported stability in the prevalence of depressive disorders between 2014 and 2018 in the population aged 15 and over (10,11).

Health surveys conducted in the United States (8) and Canada (19) found no variation in the prevalence of psychiatric morbidity between the 2000s and 2010s. A survey conducted in Australia reported an increase in prevalence of severe psychological distress between 2007 and 2017 (7). In Japan, an increase in prevalence of severe psychological distress was found, while moderate distress remained stable between 2007 and 2016 (9). In Spain, there was a reduction in the overall prevalence of mental disorders between 2006 and 2012 (6).

The results of this study, indicating a reduction in the overall prevalence of common mental disorders between 2003 and 2015, corroborate the findings of two systematic reviews that found no evidence of a global increase in the prevalence of depressive and anxiety disorders (13,14). One of the reviews mentioned above conducted post hoc analysis with studies that had psychological distress as their outcome. That review indicated a trend towards an increase in the prevalence of this psychiatric morbidity, differing from our investigation (13). Since the majority of studies included in these reviews come from high-income countries, the generalizability of these results is limited.

With regard to the economic context during the three editions of the ISA-Capital Survey, it is noteworthy that although the end of the period analyzed in this investigation corresponds to the beginning of the recession, it was predominantly characterized by positive socioeconomic changes, such as reduction of poverty, increase in formal jobs, better wages and expansion of income transfer programs (20). This combination of factors over time, linked to the expansion of the basic and psychosocial care network in the metropolitan region of São Paulo city (21), may have contributed to the reduction in the overall prevalence of common mental disorders.

Another relevant finding of this work was its identifying that when compared to males sex, there was a substantial reduction in the prevalence of common mental disorders among females between 2003 and 2015, in the 20-59 age range. The increase in schooling, income and participation in the formal job market, seen more prominently among women in recent decades, may have contributed to reducing the gender disparity in the prevalence of these disorders over time (22). More unpaid work time (23) and a greater number of children (24) are associated with poorer mental health in women in this age group. It is possible that the reduction in fertility rate (25) and unpaid work (26) explain part of these findings. Linked to this set of factors and with a greater effect among younger women, as observed in this study, possible changes in gender roles over time may also contribute to explaining the reduction in the disparity between the sexes regarding prevalence of common mental disorders (27). 

Still with regard to the 20-59 age group, although there was no significant variation, the temporal pattern suggesting an increase in the prevalence of these disorders in males may be related to the beginning of the financial crisis in Brazil, as previous evidence (28) indicates that this subgroup is more vulnerable to deteriorating mental health in such contexts. In Spain, the reduction in the overall prevalence of psychiatric morbidity contrasted with its increase among males aged 18 to 64, possibly associated with the financial crisis (6). Even though our investigation does not have sufficient statistical power to detect it, it is plausible to consider the hypothesis that an increase in the prevalence of common mental disorders was already occurring in this subgroup.

Although the reduction in the prevalence of common mental disorders among females, in relation to males, reduced the disparity in this psychiatric morbidity between 2003 and 2015, it still persists, being almost double that observed among males in the final year of this analysis. Furthermore, these results should be interpreted with caution, as it is possible that this trend reversed in the following years due to the economic recession and fiscal austerity measures implemented in the period. In England, the improvement in the gender gap with regard to mental health, observed before the 2008 global crisis, was reversed and worsened after austerity policies (29).

Among the limitations of this study is the use of an instrument that, although it has good sensitivity and specificity in identifying possible cases of the outcome under analysis, it does not make it possible to establish a diagnosis. Institutionalized individuals were not included in the ISA-Capital Survey, which could lead to underestimation of prevalence. Conclusions cannot be drawn about the incidence of mental disorders in the period analyzed. After 2015, socioeconomic changes in Brazil and the COVID-19 pandemic may have had a strong impact on the mental health of the population. 

Between 2003 and 2015, prevalence of common mental disorders in São Paulo city decreased in females aged between 20 and 59 years, while it remained stable among males in the same age group and among elderly people of both sexes. These results emphasize the importance of mental health actions and monitoring the prevalence of this psychiatric morbidity among the population, considering differences according to sex and age group.
