# Supplementary material for: Common mental disorders in São Paulo city, 2003-2015: population-based serial cross-sectional panel studies
Source: Epidemiol Serv Saude. 2025 May 9;34:e20240048. doi: 10.1590/S2237-96222025v34.20240048.en (PMC12064162; doi:10.1590/S2237-96222025v34.20240048.en)
Supplement: Translation

# Translation: Transtornos mentais comuns no município de São Paulo, 2003-2015: painel de estudos transversais seriados de base populacional

**DOI:** 10.1590/S2237-96222025v34.20240048.pt

**Authors:** Thiago Pestana Pinto, Camila Nascimento Monteiro, Moises Goldbaum, Chester Luiz Galvão César, Paulo Rossi Menezes

**Affiliations:** 1 Universidade de São Paulo, Faculdade de Saúde Pública, São Paulo, SP, Brasil; 2 Hospital Sírio-Libanês, São Paulo, SP, Brasil; 3 Universidade de São Paulo, Faculdade de Medicina, São Paulo, SP, Brasil

**Keywords:** Transtornos Mentais, Saúde Mental, Inquéritos Epidemiológicos, Estudos Transversais Seriados, Epidemiologia

## Abstract

**Objetivo:**

Descrever a evolução temporal dos transtornos mentais comuns entre indivíduos com 20 anos ou mais residentes na área urbana do município de São Paulo.

**Métodos:**

Painel de estudos transversais seriados de base populacional com dados das edições de 2003, 2008 e 2015 do Inquérito de Saúde do Município de São Paulo. A presença de transtornos mentais comuns foi mensurada por meio do *self reporting questionnaire*-20. Utilizando-se regressão de Poisson, foram estimadas razões de prevalências (RP) não ajustadas e ajustadas e os intervalos de confiança de 95% (IC95%), para verificar diferenças na prevalência de transtornos mentais comuns entre os anos.

**Resultados:**

O total de participantes nesta investigação foi de 1.565 em 2003, 2.019 em 2008 e 3.169 em 2015. Na análise ajustada, verificou-se redução na prevalência de transtornos mentais comuns entre 2003 e 2015 nas pessoas do sexo feminino com idade entre 20 e 59 anos (RP 0,68; IC95% 0,56; 0,84). No mesmo período, não houve variação significativa entre indivíduos de 20 a 59 anos do sexo masculino (RP 1,25; IC95% 0,86; 1,82), assim como entre aqueles com 60 anos ou mais do sexo feminino (RP 0,95; IC95% 0,69; 1,32) e masculino (RP 0,68; IC95% 0,44; 1,07).

**Conclusão:**

Entre 2003 e 2015, a prevalência de transtornos mentais comuns no município de São Paulo diminuiu em pessoas do sexo feminino com idade entre 20 e 59 anos, enquanto permaneceu estável nos demais subgrupos analisados. Esses achados mostram a importância de ações de saúde mental e do monitoramento da prevalência dessa morbidade psiquiátrica na população, considerando diferenças por sexo e faixa etária.

## Introdução

Os problemas de saúde mental representam uma das principais causas de incapacidade em todo o mundo (1). Entre esses agravos de saúde, os transtornos mentais comuns, caracterizados por um conjunto de sintomas depressivos, de ansiedade e queixas somáticas, constituem uma morbidade psiquiátrica frequente que, embora não necessariamente preencha critérios definidos pelos manuais diagnósticos para algum transtorno mental específico, também pode impactar fortemente a capacidade funcional (2).

No Brasil, estudos populacionais em diferentes centros urbanos reportaram elevadas prevalências desses transtornos, variando entre 15% e 28% (3-5). Apesar dessas evidências, há uma lacuna nos dados sobre a evolução temporal da prevalência dessa morbidade.

Considerando-se o período pré-pandemia da covid-19, de 2001 a 2019, resultados divergentes têm sido reportados em análises de tendência de problemas de saúde mental, como transtornos depressivos, de ansiedade, sofrimento psicológico e outros transtornos (6-12). Por exemplo, enquanto no Brasil (12) foi identificado aumento na prevalência de sintomas depressivos entre 2013 e 2019, no Peru (10) essa prevalência permaneceu estável entre 2014 e 2018. Já na Espanha, entre 2006 e 2016, observou-se redução nos transtornos mentais comuns entre mulheres e um aumento entre homens mais jovens. Essas informações são menos precisas para países de baixa e média renda, devido à quantidade limitada de estudos epidemiológicos seriados de base populacional abordando o tema nessas regiões, conforme apontam revisões sistemáticas prévias (13,14).

Investigar a distribuição dos transtornos mentais ao longo do tempo pode fornecer informações relevantes para o planejamento e monitoramento das ações de saúde mental. O presente estudo teve como objetivo descrever a evolução temporal (2003-2015) da prevalência de transtornos mentais comuns em adultos residentes na zona urbana do município de São Paulo.

## Métodos

### Delineamento

Trata-se de um painel de estudos transversais seriados de base populacional, realizado com dados coletados nas edições de 2003, 2008 e 2015 do Inquérito de Saúde do Município de São Paulo (ISA-Capital).

### Contexto

Realizado periodicamente, o ISA-Capital investiga aspectos relacionados às condições de vida, saúde e utilização dos serviços de saúde na população residente na área urbana do município de São Paulo (15).

### Participantes

Foram incluídos neste estudo indivíduos com 20 anos ou mais que responderam ao bloco de saúde mental, selecionados nas três edições do ISA-Capital por meio de amostragem por conglomerados em dois estágios: setores censitários e domicílios. A quantidade sorteada de setores censitários foi de 60 em 2003, 70 em 2008 e 150 em 2015. As taxas de resposta foram semelhantes nos três anos: 75% em 2003, 76% em 2008 e 74% em 2015. Para corrigir efeitos de não resposta, foram levados em conta percentuais de perdas de 20% em 2003 e 2008, e de 26% em 2015. Como a população-alvo desta investigação foram adultos, utilizaram-se apenas os dados referentes aos domínios demográficos de 20 a 59 anos e de 60 anos ou mais dos três anos do inquérito.

### Variáveis

O desfecho foi a presença de transtornos mentais comuns, avaliada por meio do *self-reporting questionnaire* (16), presente no bloco de saúde emocional dos questionários aplicados nos três anos do ISA-Capital. Considerando-se uma avaliação de desempenho mais recente (17) desse instrumento em adultos, que apresentou sensibilidade de 86% e especificidade de 89%, adotou-se o ponto de corte de 8 respostas afirmativas para ambos os sexos, ou seja, participantes que responderam “sim” para 8 ou mais dos 20 itens foram considerados com transtornos mentais comuns.

Também foram utilizadas as seguintes variáveis sociodemográficas: sexo (masculino, feminino); faixa etária (em anos: 20-59 e ≥60); raça/cor da pele (branca, não branca); situação conjugal (casado ou união estável, solteiro, separado ou divorciado, viúvo); escolaridade (em anos:≤3, 4-11, ≥12); renda mensal familiar *per capita* (em salários mínimos <1, 1-4 e >4); e trabalho remunerado ou voluntário (sim, não).

### Fonte de dados e mensuração

A coleta de dados de cada inquérito foi realizada por meio de questionários estruturados em blocos temáticos, aplicados durante entrevistas nos domicílios dos participantes.

### Controle de viés

Os entrevistadores foram treinados previamente e acompanhados ao longo de todo o período do trabalho de campo. Para verificar a qualidade dos dados em cada ano do inquérito, realizaram-se entrevistas adicionais, por telefone ou no domicílio, em amostra aleatória com 5% dos participantes.

### Tamanho do estudo

O cálculo do tamanho da amostra nas três edições do ISA-Capital considerou um cenário equivalente à máxima variabilidade na frequência dos eventos estudados (proporção de 0,50), com efeito de delineamento de 1,5 e nível de confiança de 95%. O erro de amostragem foi de 6% em 2003 e 2008, e de 10% em 2015. Em 2003 e 2008, foram considerados oito domínios demográficos na amostragem total, a saber: <1 ano; 1-11 anos; 12-19 anos, masculino e feminino; 20-59 anos, masculino e feminino; e ≥60 anos, masculino e feminino. No ISA-Capital de 2015, os domínios foram: 12-19 anos, masculino e feminino; 20-59 anos, masculino e feminino; e ≥60 anos, masculino e feminino. A amostra final foi de 3.357 em 2003, 3.271 em 2008 e 4.043 em 2015. Quando considerados apenas os indivíduos com 20 anos ou mais, a amostra foi de 1.667 em 2003, 2.086 em 2008 e 3.169 em 2015. Informações adicionais sobre aspectos metodológicos das três edições do ISA-Capital podem ser consultados em publicações anteriores (15,18).

### Métodos estatísticos

Foram realizadas análises descritivas pelas variáveis sociodemográficas. As prevalências gerais e estratificadas por sexo de transtornos mentais comuns foram estimadas tanto para a amostra total quanto por idade (20-59 e ≥60 anos), em cada ano.

A análise da evolução temporal foi elaborada a partir da comparação das prevalências de transtornos mentais comuns na população geral e nos subgrupos de sexo e faixa etária, entre 2003 e 2008, assim como entre 2003 e 2015. Para isso, foram padronizados nomes e categorias das variáveis nos bancos de dados originais dos inquéritos. Em seguida, foram criados dois bancos de dados unificados, um de 2003 e 2008 e outro de 2003 e 2015, considerando-se o processo de amostragem dos diferentes anos no ajuste dos pesos amostrais nesses bancos unificados. 

Para analisar a magnitude das diferenças de prevalências de transtornos mentais comuns entre os anos, estimaram-se razões de prevalências (RP) não ajustadas e ajustadas e intervalos de confiança de 95% (IC95%) por meio de modelos de regressão de Poisson. As RP foram ajustadas por idade, sexo, raça/cor da pele, situação conjugal, escolaridade, renda e trabalho para verificar se as diferenças na prevalência de transtornos mentais comuns, entre as edições do inquérito, eram independentes das outras variáveis da estrutura sociodemográfica. Nos modelos estratificados por sexo, fez-se o ajuste para as variáveis idade, raça/cor da pele, situação conjugal, escolaridade, renda e trabalho.

Foi analisado o comportamento da diferença da prevalência de transtornos mentais comuns entre os sexos na população total e por faixa etária (20-59 e ≥60 anos). A magnitude dessa diferença foi apresentada por meio de RP não ajustadas e ajustadas por idade, raça/cor da pele, situação conjugal, escolaridade, renda e trabalho, para cada ano.

Foi calculada interação entre as variáveis sexo e ano de inquérito, nos modelos ajustados por ano de inquérito, idade, sexo, raça/cor da pele, situação conjugal, escolaridade, renda e trabalho, sendo apresentados os p-valores dessas análises de interação.

A significância estatística foi avaliada por meio do teste de Wald, adotando-se nível de significância de 5%. As observações com dados faltantes foram excluídas, e as análises foram realizadas no software Stata 13 utilizando-se o módulo svy para dados obtidos por amostragem complexa.

## Resultados

O total de indivíduos incluídos neste estudo foi de 1.565 em 2003, 2.019 em 2008 e 3.169 em 2015. A distribuição percentual do sexo entre os anos manteve-se estável, sendo a maioria do sexo feminino (54,9% em 2003 versus 53,7% em 2015). Observou-se aumento na proporção de pessoas com 60 anos ou mais (16,0% em 2003 vs. 18,5% em 2015). Houve diminuição na proporção de pessoas autodeclaradas brancas (67,4% em 2003 vs. 52,7% em 2015) e aumento na faixa de renda inferior (38,2% em 2003 vs. 54,5% em 2015) (Tabela 1).

**Tabela 1:** Frequências e intervalos de confiança de 95% (IC95%) da população de adultos pelas características sociodemográficas e ano de inquérito. São Paulo, 2003 (n=1.667), 2008 (n=2.086) e 2015 (n=3.184)

Variáveis	n	2003, % (IC95%)	n	2008, % (IC95%)	n	2015, % (IC95%)
Sexo						
Masculino	803	45,1 (42,1; 48,1)	848	46,3 (44,3; 48,3)	1.340	46,3 (44,6; 48,0)
Feminino	864	54,9 (51,9; 57,9)	1.238	53,7 (51,7; 55,7)	1.844	53,7 (52,0; 55,4)
**Faixa etária**						
20-59	795	84,0 (81,6; 86,2)	1.162	83,7 (81,2; 85,9)	2.165	81,5 (79,3; 83,5)
≥60	872	16,0 (13,8; 18,4)	924	16,3 (14,1; 18,8)	1.019	18,5 (16,5; 20,7)
**Raça/cor da pele**						
Branca	1.077	67,4 (61,7; 72,6)	1.311	62,2 (57,2; 66,9)	1.632	52,7 (48,8; 56,5)
Não branca	542	32,6 (27,4; 38,3)	770	37,8 (33,1; 42,8)	1.531	47,3 (43,5; 51,2)
**Situação conjugal**						
Casado ou união estável	969	58,1 (54,3; 61,8)	1.181	59,4 (56,3; 62,5)	1.820	59,0 (56,5; 61,6)
Solteiro	272	27,0 (23,8; 30,5)	390	25,4 (22,6; 28,4)	712	25,9 (23,9; 28,0)
Separado ou divorciado	124	7,3 (5,83; 9,21)	187	8,3 (6,80; 10,1)	308	8,7 (7,60; 9,91)
Viúvo	274	7,6 (6,24; 9,18)	328	6,9 (5,80; 8,23)	333	6,4 (5,57; 7,32)
**Escolaridade** (anos)						
≥12	231	23,9 (18,1; 30,9)	305	24,2 (19,3; 29,9)	686	28,6 (24,8; 32,7)
4-11	959	61,9 (57,0; 66,6)	1.365	65,6 (61,4; 69,6)	1.935	59,7 (56,2; 63,1)
≤3	450	14,2 (11,4; 17,5)	413	10,2 (8,24; 12,6)	545	11,7 (10,2; 13,4)
**Renda familiar** (**salários mínimos**)						
>4	233	19,5 (13,0; 28,3)	219	14,8 (10,6; 20,2)	254	8,9 (7,04; 11,3)
1-4	745	42,3 (37,1; 47,7)	1.023	47,5 (43,2; 51,8)	1.141	36,6 (32,8; 40,5)
<1	689	38,2 (32,0; 44,7)	844	37,7 (32,7; 43,1)	1.789	54,5 (50,2; 58,7)
**Trabalho remunerado**/voluntário						
Sim	654	57,5 (54,1; 60,9)	975	65,3 (62,3; 68,1)	1.787	64,2 (61,9; 66,5)
Não	995	42,5 (39,1; 45,9)	1.110	34,7 (31,9; 37,7)	1.385	35,8 (33,5; 38,1)

Em 2003, a prevalência geral de transtornos mentais comuns foi 22,0% (IC95% 18,8; 25,^7^); houve redução significativa tanto em 2008 (RP 0,76 ; IC95% 0,61; 0,94) quanto em 2015 (RP 0,76; IC95% 0,62; 0,93). Após ajuste para o conjunto de variáveis da estrutura sociodemográfica, tais diferenças mantiveram-se estatisticamente significantes, tanto no período 2003-2008 (RP 0,81; IC95% 0,67; 0,97) quanto no período 2003-2015 (RP 0,80; IC95% 0,67; 0,95).

Entre 20 a 59 anos, em ambos os sexos, observou-se diferenças significativas nas prevalências de transtornos mentais comuns, entre 2003 e 2008 (RP 0,80; IC95% 0,65; 0,97) e entre 2003 e 2015 (RP 0,79; IC95% 0,65; 0,96). Em idosos não foram observadas mudanças significativas na prevalência de transtornos entre 2003 e 2008 (RP 0,86; IC95% 0,70; 1,05) e entre 2003 e 2015 (RP 0,89; IC95% 0,67; 1,18) (Tabela 2).

**Tabela 2:** Prevalências, razões de prevalências (RP) e intervalos de confiança de 95% (IC95%) de transtornos mentais comuns por sexo, faixa etária e ano de inquérito. São Paulo, 2003 (n=1.565), 2008 (n=2.019) e 2015 (n=3.169)

Grupo etário e sexo	2003	2008	2015	2003-2008				2003-2015			
	% (IC95%)	% (IC95%)	% (IC95%)	RP bruta (IC95%)	p-valor	RP ajustada (IC95%)	p-valor	RP bruta (IC95%)	p-valor	RP ajustada (IC95%)	p-valor
≥**20 anos**											
Total	22,0 (18,8; 25,7)	16,8 (14,9; 18,8)	16,8 (15,3; 18,5)	0,76 (0,61; 0,94)	0,012	0,81 (0,67; 0,97)	0,021	0,76 (0,62; 0,93)	0,009	0,8 (0,67; 0,95)	0,011
Masculino	9,8 (8,7; 9,2)	8,0 (6,0; 10,6)	10,4 (8,8; 12,4)	0,82 (0,56; 1,21)	0,322	0,86 (0,60; 1,25)	0,439	1,07 (0,78; 1,47)	0,672	1,14 (0,83; 1,56)	0,42
Feminino	32 (27,2; 37,1)	24,3 (21,5; 27,2)	22,4 (20,0; 24,9)	0,75 (0,60; 0,93)	0,011	0,80 (0,66; 0,96)	0,017	0,70 (0,57; 0,86)	0,001	0,72 (0,60; 0,87)	0,001
**20-59 anos**											
Total	22,3 (18,6; 26,6)	16,6 (14,6; 18,9)	16,8 (15,2; 18,7)	0,74 (0,59; 0,94)	0,016	0,80 (0,65; 0,97)	0,026	0,75 (0,60; 0,94)	0,014	0,79 (0,65; 0,96)	0,018
Masculino	8,8 (6,3; 12,1)	7,8 (5,6; 10,8)	10,6 (8,8; 12,8)	0,89 (0,56; 1,40)	0,606	0,92 (0,59; 1,43)	0,703	1,21 (0,83; 1,75)	0,324	1,25 (0,86; 1,82)	0,235
Feminino	33,6 (28,1; 39,7)	24,5 (21,5; 27,8)	22,5 (19,9; 25,4)	0,73 (0,58; 0,92)	0,008	0,77 (0,63; 0,94)	0,012	0,67 (0,53; 0,84)	0,001	0,68 (0,56; 0,84)	<0,001
≥**60 anos**											
Total	20,5 (17,4; 23,9)	17,4 (15,0; 20,1)	16,8 (13,4; 20,8)	0,85 (0,68; 1,06)	0,150	0,86 (0,70; 1,05)	0,132	0,82 (0,62; 1,08)	0,152	0,89 (0,67; 1,18)	0,405
Masculino	15,6 (11,5; 20,9)	9,3 (6,2; 13,6)	9,5 (6,8; 13,0)	0,59 (0,36; 0,98)	0,041	0,62 (0,39; 1,01)	0,054	0,60 (0,39; 0,95)	0,028	0,68 (0,44; 1,07)	0,095
Feminino	23,7 (19,2; 28,9)	23,1 (19,4; 27,2)	21,7 (17,0; 27,4)	0,97 (0,74; 1,27)	0,846	0,91 (0,74; 1,20)	0,621	0,82 (0,67; 1,26)	0,594	0,95 (0,69; 1,32)	0,778

Entre participantes do sexo masculino na faixa etária de 20 a 59 anos, não houve variação significativa na prevalência de transtornos mentais comuns entre 2003 e 2008 (RP 0,92; IC95% 0,59; 1,43) e entre 2003 e 2015 (RP 1,25; IC95% 0,86; 1,82). Da mesma forma, não foram observadas mudanças significativas entre homens idosos nos períodos de 2003-2008 (RP 0,62; IC95% 0,39; 1,01) e de 2003-2015 (RP 0,68; IC95% 0,44; 1,07). Entre as mulheres de 20 a 59 anos, observou-se redução significativa na prevalência de transtornos mentais comuns tanto entre 2003 e 2008 (RP 0,77; IC95% 0,63; 0,94) quanto entre 2003 e 2015 (RP 0,68; IC95% 0,56; 0,84). Por outro lado, entre as mulheres idosas, não foram identificadas mudanças nos períodos de 2003-2008 (RP 0,91; IC95% 0,74; 1,20) e de 2003-2015 (RP 0,95; IC95% 0,69; 1,07) (Tabela 2) e (Figura 1).

**Figura 1:** Prevalência e intervalos de confiança de 95% (IC95%) de transtornos mentais comuns por sexo, faixas etárias e ano de realização do inquérito. São Paulo, 2003 (n=1.565), 2008 (n=2.019) e 2015 (n=3.169)

Especialmente na faixa etária de 20 a 59 anos, observou-se redução na magnitude da diferença da prevalência de transtornos mentais comuns entre os sexos, passando de RP 3,19 (IC95% 2,31; 4,42) em 2003 para RP 2,01 (IC95% 1,58; 2,55) em 2015. Considerando-se as edições do ISA-Capital de 2003 e 2008, não houve interação significativa entre sexo e ano de inquérito tanto na população total (p-valor 0,901), quanto nas faixas etárias de 20 a 59 anos (p-valor 0,606) e de 60 anos ou mais (p-valor 0,155). Na comparação entre 2003 e 2015, a evolução temporal de transtornos mentais comuns foi significativamente diferente entre os sexos na população geral (p-valor 0,015) e na faixa etária de 20 a 59 anos (p-valor 0,005) (Tabela 3).

**Tabela 3:** Razões de prevalências (RP) e intervalos de confiança de 95% (IC95%) de transtornos mentais comuns entre os sexos, por faixa etária e ano de inquérito. São Paulo, 2003 (n=1.565), 2008 (n=2.019) e 2015 (n=3.169)

Grupo etário e sexo	2003		2008		2015		Interação sexo##ano	
	RP bruta (IC95%)	RP ajustada (IC 95%)	RP bruta (IC95%)	RP ajustada (IC 95%)	RP bruta (IC95%)	RP ajustada (IC 95%)	2003-2008	2003-2015
≥**20 anos**								
Masculino	1,00	1,00	1,00	1,00	1,00	1,00	0,901	0,015
Feminino	3,28 (2,53; 4,23)	2,78 (2,12; 3,64)	3,03 (2,26; 4,05)	2,88 (2,08; 4,01)	2,14 (1,75; 2,62)	2,02 (1,63; 2,51)		
**20-59 anos**								
Masculino	1,00	1,00	1,00	1,00	1,00	1,00	0,606	0,005
Feminino	3,81 (2,81; 5,18)	3,19 (2,31; 4,42)	3,13 (2,22; 4,43)	3,02 (2,03; 4,50)	2,12 (1,69; 2,65)	2,01 (1,58; 2,55)		
≥**60 anos**								
Masculino	1,00	1,00	1,00	1,00	1,00	1,00	0,155	0,307
Feminino	1,51 (1,03; 2,23)	1,44 (0,89; 2,30)	2,49 (1,59; 3,89)	2,03 (1,20; 3,42)	2,29 (1,62; 3,25)	1,85 (1,23; 2,78)		

## Discussão

Entre 2003 e 2015 observou-se redução significativa na prevalência de transtornos mentais comuns em mulheres de até 59 anos. Entre os homens a prevalência não se alterou, bem como entre idosos. A interação entre as variáveis sexo e ano de inquérito no grupo etário de 20 a 59 anos indica que, entre 2003 e 2015, a evolução temporal da prevalência de transtornos mentais comuns foi distinta entre os sexos, com uma diminuição significativa da prevalência nas mulheres em comparação aos homens.

Diferentemente desta investigação, observou-se aumento na prevalência de sintomas depressivos entre 2013 e 2019 na Pesquisa Nacional de Saúde (12). A recessão econômica no Brasil, em 2015 e 2016, é um importante fator a ser ponderado na comparação com a presente investigação. Conforme enfatizado pelos autores, grande parte do período analisado no referido artigo foi permeado por desaceleração econômica e recessão no país, o que explicaria essa tendência de aumento (12).

Por seu turno, também na América do Sul, um inquérito de saúde realizado no Chile reportou prevalência estável de transtornos depressivos entre 2003 e 2010 em indivíduos com 18 anos ou mais. Resultado semelhante foi identificado em um inquérito no Peru, que reportou estabilidade na prevalência de transtornos depressivos entre 2014 e 2018 na população com 15 anos ou mais (10,11).

Inquéritos de saúde realizados nos Estados Unidos (8) e no Canadá (19) não encontraram variações na prevalência de morbidade psiquiátrica entre as décadas de 2000 e 2010. Um inquérito conduzido na Austrália relatou aumento na prevalência de sofrimento psicológico grave entre 2007 e 2017 (7). No Japão, observou-se aumento na prevalência de sofrimento psicológico grave, enquanto o sofrimento moderado permaneceu estável entre 2007 e 2016 (9). Na Espanha, observou-se redução na prevalência geral de transtornos entre 2006 e 2012 (6).

Os resultados deste estudo, indicando redução na prevalência geral de transtornos mentais comuns entre 2003 e 2015, corroboram achados de duas revisões sistemáticas que não encontraram evidências de aumento global na prevalência de transtornos depressivos e de ansiedade (13,14). Em uma dessas revisões mencionadas anteriormente, foi realizada uma análise *post hoc* com estudos cujo desfecho era sofrimento psicológico que indicou uma tendência de aumento na prevalência dessa morbidade psiquiátrica, diferindo desta investigação (13). Uma vez que a maioria dos estudos incluídos nessas revisões provém de países de alta renda, a generalização desses resultados é limitada.

Em relação ao contexto econômico durante as três edições do ISA-Capital, destaca-se que embora o final do período analisado nesta investigação corresponda ao início da recessão, este foi predominantemente caracterizado por mudanças socioeconômicas positivas, como a redução da pobreza, o aumento de empregos formais, melhores salários e a expansão de programas de transferência de renda (20). Essa combinação de fatores ao longo do tempo, atrelada à expansão da rede de atenção básica e psicossocial na região metropolitana do município de São Paulo (21), pode ter contribuído para a redução da prevalência geral de transtornos mentais comuns.

Outro relevante achado deste trabalho foi identificar que em relação ao sexo masculino, houve uma redução substancial da prevalência de transtornos mentais comuns no sexo feminino entre 2003 e 2015, na faixa de 20 a 59 anos. O aumento da escolaridade, da renda e da participação no mercado formal de trabalho, observado nas últimas décadas de maneira mais proeminente entre as mulheres, pode ter contribuído para reduzir a disparidade de gênero na prevalência desses transtornos ao longo do tempo (22). Mais tempo de trabalho não remunerado (23) e maior número de filhos (24) estão associados à pior saúde mental em mulheres nessa faixa etária. É possível que a redução na taxa de fecundidade (25) e no trabalho não remunerado (26) expliquem parte desses achados. Atreladas a esse conjunto de fatores e com maior efeito entre mulheres mais jovens, como observado neste estudo, possíveis mudanças nos papéis de gênero, ao longo do tempo, também podem contribuir para explicar a redução da disparidade entre os sexos na prevalência de transtornos mentais comuns (27). 

Nessa mesma faixa etária de 20-59 anos, embora não tenha ocorrido variação significativa, o padrão temporal sugerindo aumento na prevalência desses transtornos em homens pode estar relacionado ao início da crise financeira no Brasil, já que evidências (28) prévias indicam que esse subgrupo é mais vulnerável à deterioração da saúde mental em tais contextos. Na Espanha, a redução na prevalência geral de morbidade psiquiátrica contrastou com o aumento entre homens de 18 a 64 anos, possivelmente associado à crise financeira (6). Mesmo que a presente investigação não tenha poder estatístico suficiente para detectar, é plausível considerar a hipótese de que já estivesse ocorrendo elevação na prevalência de transtornos mentais comuns nesse subgrupo.

Embora a redução na prevalência de transtornos mentais comuns entre as mulheres, em relação aos homens, tenha diminuído a disparidade dessa morbidade psiquiátrica entre 2003 e 2015, ela ainda persiste, sendo quase o dobro da observada entre homens no último ano desta análise. Além disso, esses resultados devem ser interpretados com cautela, pois é possível que essa tendência tenha se revertido nos anos seguintes devido à recessão econômica e às medidas de austeridade fiscal implementadas no período.Na Inglaterra, a melhora na disparidade de gênero em saúde mental, observada antes da crise global de 2008, foi revertida e agravada após as políticas de austeridade (29).

Entre as limitações está o uso de um instrumento que, embora tenha boa sensibilidade e especificidade na identificação de possíveis casos do desfecho analisado, não possibilita estabelecer diagnóstico. No ISA-Capital não foram incluídos indivíduos institucionalizados, podendo levar à subestimação da prevalência. Não se podem tirar conclusões acerca da incidência de transtornos mentais no período analisado. Após 2015, mudanças socioeconômicas no país e a pandemia de covid-19 podem ter impactado fortemente a saúde mental da população. 

Entre 2003 e 2015, a prevalência de transtornos mentais comuns no município de São Paulo diminuiu nas mulheres com idade entre 20 e 59 anos, enquanto permaneceu estável entre os homens da mesma faixa etária e entre idosos de ambos os sexos. Esses resultados enfatizam a importância de ações de saúde mental e do monitoramento da prevalência dessa morbidade psiquiátrica na população, considerando diferenças por sexo e faixa etária.
